# Microfluidic Endothelium for Studying the Intravascular Adhesion of Metastatic Breast Cancer Cells

**DOI:** 10.1371/journal.pone.0005756

**Published:** 2009-06-01

**Authors:** Jonathan W. Song, Stephen P. Cavnar, Ann C. Walker, Kathryn E. Luker, Mudit Gupta, Yi-Chung Tung, Gary D. Luker, Shuichi Takayama

**Affiliations:** 1 Department of Biomedical Engineering, University of Michigan, Ann Arbor, Michigan, United States of America; 2 Department of Radiology, University of Michigan, Ann Arbor, Michigan, United States of America; 3 Department of Microbiology and Immunology, University of Michigan, Ann Arbor, Michigan, United States of America; 4 Macromolecular Science and Engineering Center, University of Michigan, Ann Arbor, Michigan, United States of America; Roswell Park Cancer Institute, United States of America

## Abstract

**Background:**

The ability to properly model intravascular steps in metastasis is essential in identifying key physical, cellular, and molecular determinants that can be targeted therapeutically to prevent metastatic disease. Research on the vascular microenvironment has been hindered by challenges in studying this compartment in metastasis under conditions that reproduce *in vivo* physiology while allowing facile experimental manipulation.

**Methodology/Principal Findings:**

We present a microfluidic vasculature system to model interactions between circulating breast cancer cells with microvascular endothelium at potential sites of metastasis. The microfluidic vasculature produces spatially-restricted stimulation from the basal side of the endothelium that models both organ-specific localization and polarization of chemokines and many other signaling molecules under variable flow conditions. We used this microfluidic system to produce site-specific stimulation of microvascular endothelium with CXCL12, a chemokine strongly implicated in metastasis.

**Conclusions/Significance:**

When added from the basal side, CXCL12 acts through receptor CXCR4 on endothelium to promote adhesion of circulating breast cancer cells, independent of CXCL12 receptors CXCR4 or CXCR7 on tumor cells. These studies suggest that targeting CXCL12-CXCR4 signaling in endothelium may limit metastases in breast and other cancers and highlight the unique capabilities of our microfluidic device to advance studies of the intravascular microenvironment in metastasis.

## Introduction

Trafficking of cells through the vasculature and arrest of circulating cells on endothelium at specific sites are critical steps in a wide variety of physiologic and disease processes, including immune surveillance [1]–[3], inflammation [4]–[6], and metastatic cancer [7]–[9]. In particular, specific interactions between circulating cancer cells and vascular endothelium are proposed to control patterns of metastasis for breast [10], lung [11], and other common solid cancers [12]. However, mechanisms for organ-specific tropism of metastatic cancer cells remain poorly defined.

Identifying molecular determinants of trafficking and arrest of circulating cancer cells on endothelium at characteristic sites of metastatic disease has been limited in large part by challenges of studying the intravascular microenvironment under physiologic conditions. *In vitro* assays of cancer cells and endothelium typically are performed under static, biochemically homogeneous conditions. These static assays do not accurately model intravascular events in metastasis because blood flow alters gene expression [13], [14], mechanical properties of cells [15], [16], and cell adhesion [17], [18]. Furthermore, all cells in a culture well are exposed to the same biochemical milieu, unlike physiologic conditions where gradients of signaling molecules in different anatomic locations typically are presented to the basal surface of endothelial cells [19], [20]. By comparison, animal models reproduce key physiologic features of metastatic cancer in humans, but it is difficult to selectively manipulate and control experimental conditions in the intravascular microenvironment *in vivo*. Animal models of metastasis also are time-consuming, labor-intensive, and expensive.

We have engineered a new microfluidic model of the vasculature to overcome limitations of existing systems and advance studies of the intravascular compartment in cancer metastasis and other diseases. This microfluidic device produces defined flow rates within a range of physiologic levels and enables real-time bright field and fluorescence imaging of circulating cancer cells and microvascular endothelium. The device also allows region-specific treatment of endothelium from the basal side, recreating organ-specific localization of signaling molecules produced by stromal cells in the extravascular space. This also mimics serial interaction of circulating tumor cells with endothelia of differing potentials to promote cell adhesion and formation of metastases. To establish the utility of this microfluidic device for studying intravascular steps in metastasis, we investigated effects of chemokine CXCL12 on adhesion of circulating breast cancer cells to endothelium. High levels of CXCL12 are expressed by parenchymal cells in organs commonly affected by metastatic breast cancer, such as liver, bone, and brain [21], suggesting that gradients of CXCL12 regulate homing of disseminated breast cancer cells to endothelium in these sites. CXCL12-mediated tropism of malignant breast [21], lung [11], and other cancer cells for characteristic sites of metastatic disease has been attributed predominantly to signaling through receptor CXCR4 on tumor cells [22]. However, pro-metastatic effects of CXCL12 also may be regulated through CXCR4 on vascular endothelium [23], which upregulates and activates adhesion molecules to promote stable interactions with circulating cells [24], and/or CXCR7 [25], a newly identified second receptor for CXCL12 [26].

Using the microfluidic vasculature to selectively manipulate cancer cells and endothelium, we demonstrate that circulating breast cancer cells preferentially adhere to endothelium stimulated from the basal side with CXCL12. CXCL12 functions through CXCR4 on endothelial cells to significantly enhance adhesion of circulating breast cancer cells, independent of CXCR4 or CXCR7 on cancer cells. These results provide new insights into pro-metastatic effects of CXCL12 in the intravascular microenvironment, focusing attention on CXCL12-CXCR4 in vascular endothelium as a potential therapeutic target to block metastasis. Moreover, the research establishes this microfluidic vasculature technology as a versatile, physiologic model for future studies of the intravascular microenvironment in metastasis and other disease processes.

## Results

### Fabrication of the microfluidic device

**Figure 1:**
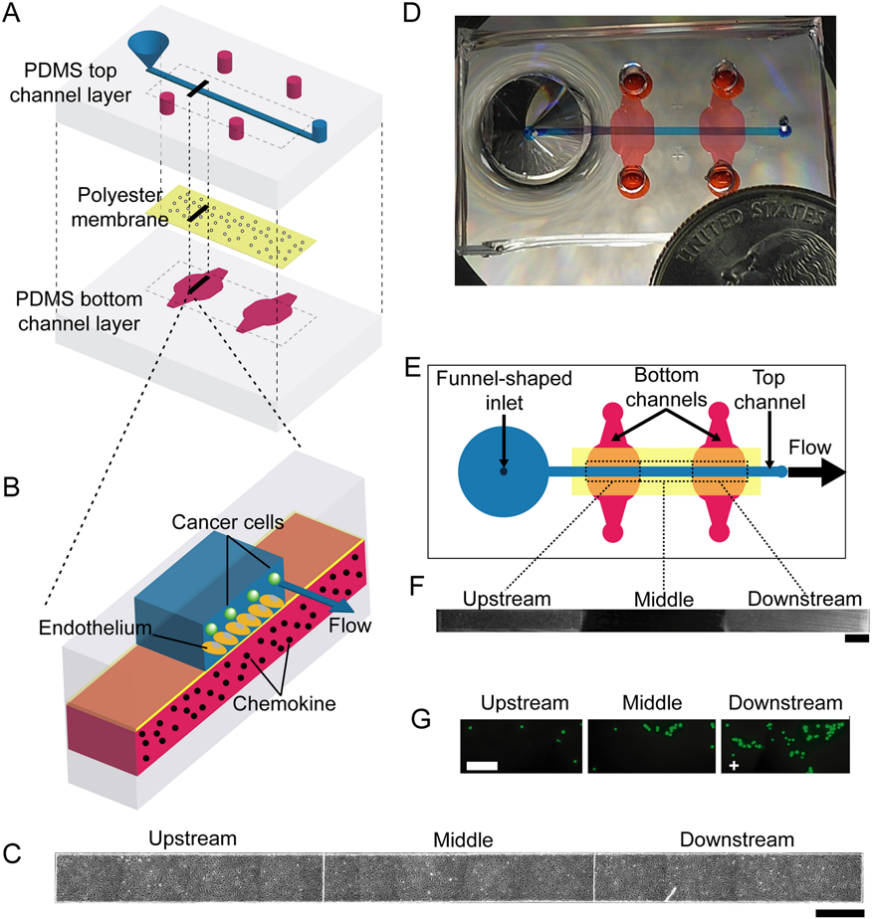
Microfluidic vasculature enables region-specific activation of endothelium under physiological flow conditions. (A) Schematic of the poly(dimethysiloxane) (PDMS) microfluidic device demonstrating multi-layer fabrication with a thin, porous polyester membrane sandwiched by the top and bottom PDMS layers. (B) Chemokine in the bottom channel activates the endothelium from the basal face while cancer cells flow in the top channel above the endothelium. (C) Phase contrast image of HDMECs cultured in the microfluidic device. The image depicts the entire length of the endothelium with the upstream, middle, and downstream regions clearly demarcated from each other. Scale bar represents 800 µm. (D) Photograph of the microfluidic device loaded with blue dye in the top channel and red dye in the two bottom channels. (E) Top view of the microfluidic device. Fluid flows away from the funnel-shaped inlet and through the top channel. (F) Validation of region-specific stimulation of endothelium. The upstream and downstream lower channels were filled with Syto 64, a fluorescent dye that stains cells. HDMECs overlying the upstream and downstream bottom channels, but not HDMECs in the middle of the channel fluoresce. Fluorescence was evident within 15 minutes, remained spatially-restricted for at least 5.5 hours, and persisted after 30 minutes of flow through the top channel. Image shows the 5.5 hour point after initial treatment. Scale bar represents 800 µm. (G) Representative fluorescent images of adhesion of 231-control cells (stably expressing GFP) onto region-specific TNF-α treated endothelium under 0.50 dyn cm^−2^ shear stress flow conditions. In these images, the downstream region was treated with TNF-α for 5 h (denoted by a ‘+’) while the upstream and middle regions were left untreated. Scale bar represents 200 µm.

The microfluidic vasculature is comprised of two poly(dimethysiloxane) (PDMS) layers sandwiching a thin, porous, and optically clear polyester membrane (Figure 1A, B). The top PDMS layer features a channel with a funnel–shaped inlet that intersects regionally distinct, perpendicularly-oriented channels in the bottom PDMS layer (Figure 1D, E). The top channel (60 µm height, 800 µm width) contains a confluent monolayer of human dermal microvascular endothelial cells (HDMECs) (Figure 1C) cultured on the polyester membrane with 400 nm pores to permit diffusion of biomolecules between the top and bottom channels (Figure 1B) as used in traditional Transwell inserts for cell culture. The regions of the top channel that intersect one of the bottom channels (60 µm height) are designated as either the upstream or downstream region (Figure 1C, F), depending on the location relative to the funnel-shaped inlet. By activating endothelia over only one of the regions, the device reproduces serial interactions of tumor cells with endothelia of differing metastasis-supporting potential as occur physiologically.

**Figure 2:**
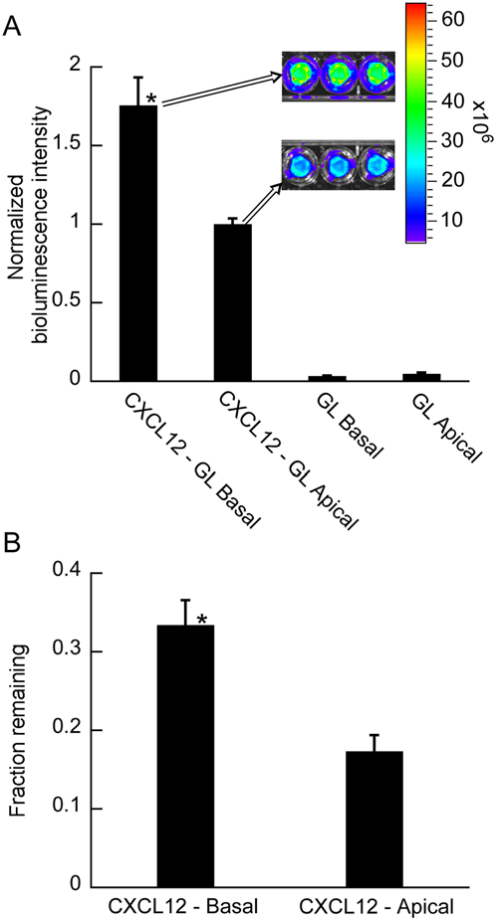
Enhanced binding of CXCL12 presented to the basal versus apical surface of endothelium mediates increased adhesion of cancer cells. (A) HDMEC cells 2 cultured on transwell inserts were incubated with ≈ 40 ng ml^−1^ CXCL12-GL added to either the top (apical) or bottom (basal) compartment for 5 hours. Control wells were incubated with equal amount of unfused GL (based on ioluminescence) added to either the top or bottom compartment. Cell-associated luciferase activity from CXCL12-GL or GL was quantified by bioluminescence imaging. Data are mean values combined from two independent experiments (n = 4, 5 for each condition). Inset shows representative bioluminescence images of CXCL12-GL associated with endothelial cells. Images are presented as a pseudocolor display with red and blue depicting the highest and lowest levels of photons, respectively. Note, 40 ng ml^−1^ is the highest concentration of CXCL12-GL that we can collect. (b) HDMECs in transwells were treated with 100 ng ml^−1^ CXCL12 either apically or basally. Following incubation with 231-control cancer cells and subsequent washing to remove non-adherent cells, approximately twice as many cancer cells remain adhered onto endothelium treated with CXCL12 basally versus apically. The fraction of cells that remain adhered was calculated from the number of cancer cells added into empty wells from the same cell suspension. n = 36 for each condition. *, p<0.05.

This setup enables simultaneous and distinct localization of chemokines on the basal face and flow on the apical face of the endothelium (Figure 1B). This capability provides proper modeling of the directionality of chemokine stimulation (basal originating instead of apical) at the vasculature of target sites for metastasis; a directionality previously shown to differentially regulate endothelial function [27]. Comparisons with the specific endothelial cells used in this study also showed that almost twice as much CXCL12 binds to the endothelium when treated from below or basally compared to apically (Figure 2A) (p<0.05). In addition, approximately twice as many MDA-MB-231 (231-control) breast cancer cells adhere onto the endothelium when the endothelium is treated with CXCL12 basally compared to apically. (Figure 2B) (p<0.05).

### Region selective treatment of the microfluidic endothelium

HDMECs in the upstream and downstream region can be selectively treated with biomolecules from the lower channels, allowing direct comparison of cancer cell adhesion onto stimulated versus non-stimulated regions of the endothelium (Figure 1G). We observed, for example, significantly greater adhesion of circulating MDA-MB-231 (231-control) breast cancer cells onto the region of the endothelium treated with the pro-inflammatory cytokine tumor necrosis factor-α (TNF-α) compared to the untreated region at shear stress levels of 0.50 dyn cm^−2^ (Figure 1G, Supplemental Figure S1A) and 2.50 dyn cm^−2^ (Supplemental Figure S1B) (p<0.02). Analysis of adhesion selectivity, defined as the ratio of 231-control cells adhering to the TNF-α treated region of the endothelium relative to the untreated region, provides further useful insights. Here, we found that the adhesion selectivity towards TNF-α treated endothelium was statistically greater at 2.50 dyn cm^−2^ compared to both 0 and 0.50 dyn cm^−2^ (Supplemental Figure S1C (p<0.05) while the total number of cells adhered decreased (Supplemental Figure S1A, B).

### Adhesion of cancer cells stably expressing CXCR4 or CXCR7 onto microfluidic endothelium

**Figure 3:**
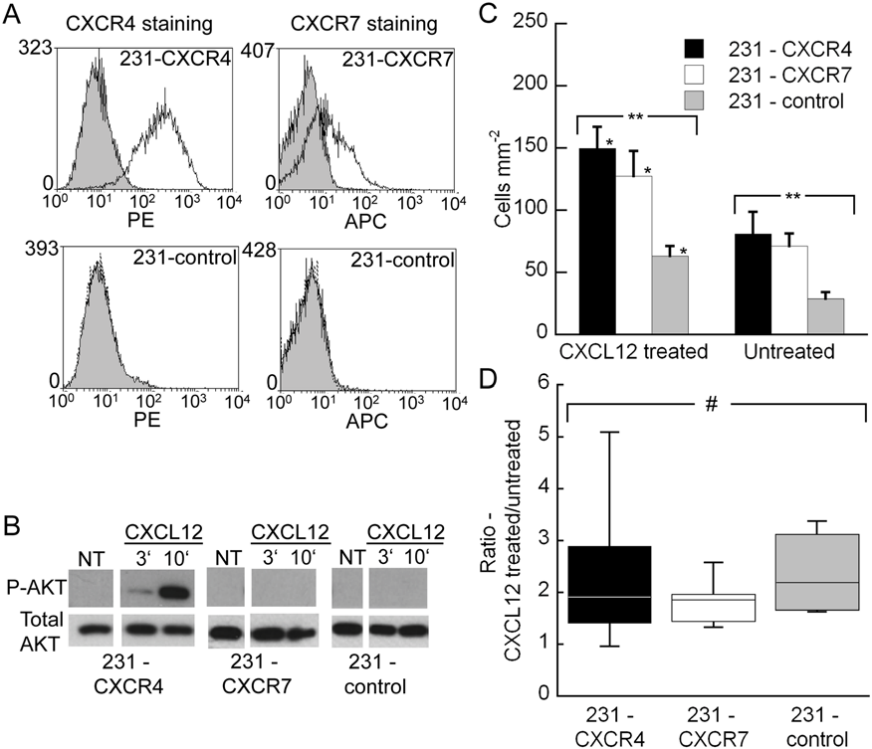
Endothelial cells mediate CXCL12-dependent enhancement in adhesion of cancer cells. (A) Flow cytometry with antibodies to CXCR4 (12G5) or CXCR7 (11G8) shows expression in stably transduced cell lines and absence of these receptors in 231-control cells. Filled histogram, isotype antibody control; open histogram, antibody stain. Isotype control staining overlapped with unstained cells (not shown on the plots). (B) CXCL12-dependent activation of AKT in 231 cells determined by Western Blot. Breast cancer cell lines were cultured overnight in medium containing 0.5% serum and then treated with 100 ng ml^−1^ CXCL12 for 3 min, 10 min, or no CXCL12 (NT). Cell lysates were probed for phosphorylation of AKT at serine 473. Blots then were stripped and probed for total AKT as a loading control. Similar results were obtained with 300 ng ml^−1^ and incubations extending through 30 minutes. (C) Adhesion of 231-CXCR4, 231-CXCR7, and 231-control cancer cells onto CXCL12 treated versus untreated endothelium under 0.50 dyn cm^−2^ shear flow conditions. For all three cancer cell-types, adhesion selectivity was towards the CXCL12 treated region of the endothelium over the corresponding untreated region (*). The level of adhesion of the three different cancer cell-types was statistically different on both the CXCL12 treated and untreated endothelial regions (**). Data are expressed as mean+s.e.m. (D) Adhesion selectivity of cancer cells towards the CXCL12 treated endothelial region over the untreated region under 0.50 dyn cm^−2^ flow conditions. Adhesion selectivity to CXCL12-stimulated endothelium was comparable for all cancer cell lines, regardless of expression of CXCR4 or CXCR7. #, p = 0.48. Statistical significance as determined by Mann-Whitney (*, p<0.05) for pair wise comparison and Kruskal-Wallis (**, p<0.05) for comparison of more than two groups.

**Figure 4:**
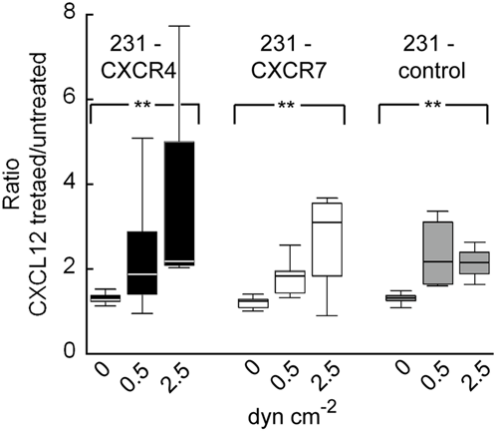
Adhesion selectivity of cancer cells onto the CXCL12 treated endothelial region over the untreated region at different flow conditions. The boxplots were grouped together by cancer cell type at 0 (or static), 0.50, and 2.50 dyn cm^−2^ flow conditions. For each cell type, the adhesion selectivity was statistically different for the different flow conditions (**, p<0.05) with the selectivity consistently increasing from static to flow conditions. n = 4–6 for each condition.

Previous reports have shown that CXCR4 expression in cancer cells promotes metastasis to distant organs such as the lung [21], [28] and that CXCR7 expression in cancer cells enhances adhesion onto endothelium under static conditions [26]. We compared 231-control cells to MDA-MB-231 cells stably expressing CXCR4 (231-CXCR4) or CXCR7 (231-CXCR7) (Figure 3A, B) to assess effects of CXCL12 chemokine receptors on cancer cells in mediating adhesion onto the microfluidic endothelium. For each of the three cancer cell types, adhesion preference was towards CXCL12-treated endothelium over the corresponding untreated endothelium in the same device (Figure 3C) (p<0.05). For all three cancer cell types, adhesion selectivity towards CXCL12-treated endothelium increased with increasing flow rates among 0, 0.50, and 2.50 dyn cm^−2^ (Figure 4) (p<0.05). The level of adhesion of the three cancer cell types was statistically different on both the CXCL12-treated and untreated endothelium (Figure 3C) (p<0.05) with the adhesion of 231-CXCR4 and 231-CXCR7 cells being significantly greater than 231-control cells (p<0.05). While CXCR4 or CXCR7 expression on circulating breast cancer cells increased adhesion to endothelium (Figure 3C), CXCL12-dependent enhancements in adhesion or adhesion selectivity were, surprisingly, statistically the same (Figure 3D) (p = 0.48) and therefore independent of expression of CXCR4 or CXCR7 on cancer cells.

### CXCR4 on endothelial cells mediates adhesion of cancer cells

**Figure 5:**
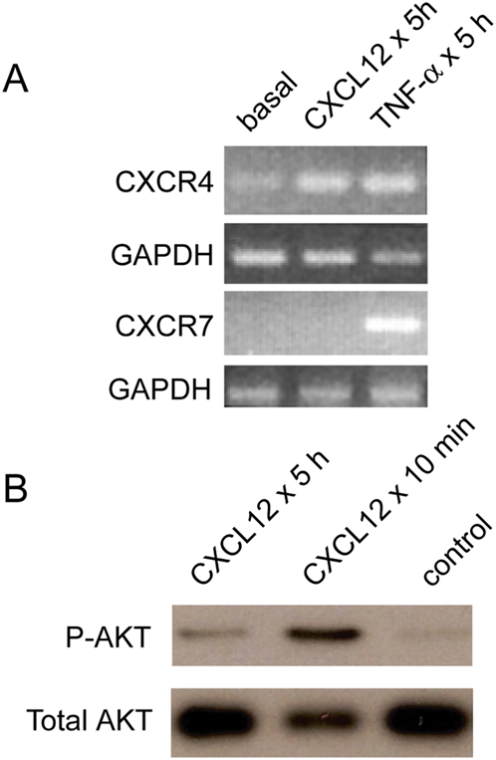
Characterization of CXCL12-mediated response in HDMECs. (A) Chemokine receptor expression in HDMECs determined by reverse transcription PCR (RT-PCR). HDMECs show expression of CXCR4 but not CXCR7 under basal conditions. CXCR4 on HDMECs is upregulated compared to basal conditions by CXCL12 and TNF-α. CXCR7 is upregulated by TNF-α only. RT-PCR for the housekeeping gene GAPDH confirms equivalent loading and intact RNA for all samples. (B) CXCL12 activates AKT in HDMECs as determined by Western Blot. There is CXCL12-dependent activation of AKT that remains greater than control endothelium through 5 hours.

To evaluate this surprising lack of difference in adhesion selectivity, we next turned our attention to how CXCL12 and receptors CXCR4 and CXCR7 on the vascular endothelia, rather than on cancer cells, may be key determinants in the intravascular adhesion step of metastasis. CXCL12 signaling in endothelial cells is known to upregulate and activate adhesion molecules, promoting stable interactions with circulating cells [24]. CXCL12 upregulated CXCR4 in HDMECs whereas TNF-α upregulated both CXCR4 and CXCR7 (Figure 5A). HDMECs treated with CXCL12 activated AKT, a known downstream effector of CXCR4 [29], showing intact signaling in these cells (Figure 5B).

**Figure 6:**
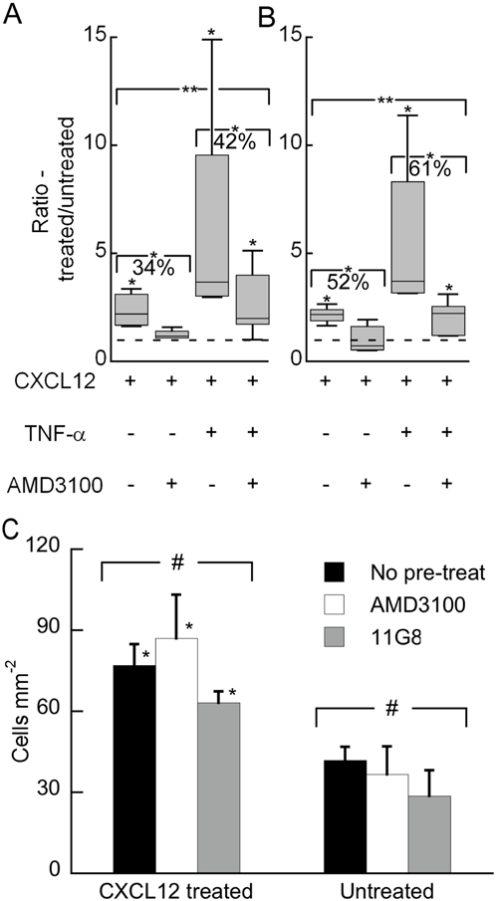
Endothelial-CXCR4 dependent adhesion of cancer cells. (A) Adhesion selectivity of 231-control cancer cells towards regions of the endothelium treated with different combinations of CXCL12, TNF-α and the CXCR4 inhibitor AMD3100 for 5 hours under 0.50 dyn cm^−2^ flow conditions (*). The levels of adhesion onto the differently treated regions were statistically different (**). AMD3100 in the presence of CXCL12 decreased adhesion by 34% relative to the CXCL12 only condition (*). AMD3100 in the presence of CXCL12 and TNF-α decreased adhesion 42% relative to CXCL12+TNF-α (*). The dotted line represents a ratio of 1 or no adhesion selectivity. (B) Same as (A) except the experiments were performed under 2.50 dyn cm^−2^ flow conditions. n = 4–6 for each condition. (C) Adhesion of 231-control cancer cells is not affected by pre-treatment with chemokine receptor inhibitors. 231-control cells were pre-treated for 5 h with either an inhibitor for CXCR4 (AMD3100) or an anti-CXCR7 antibody (11G8) and compared to no pre-treatment. The level of adhesion was statistically the same for all three conditions onto either CXCL12 treated (5 h) or untreated endothelium (#, p>0.34). For all 231-control cell pre-treatment conditions, the level of adhesion was greater onto CXCL12 treated versus untreated endothelium (*). n = 5–6 for each condition.

We region selectively treated this endothelium with various combinations of CXCL12, TNF-α and AMD3100, a specific inhibitor of CXCL12 binding to CXCR4 but not CXCR7 [30] To eliminate confounding effects from cancer cell signaling we focused on 231-control breast cancer cells, which do not express CXCR4 or CXCR7, bind bioluminescent chemokine, or signal in response to CXCL12 (Figure 3A, B) (Luker et al, manuscript In Press). There was preferential adhesion to the treated endothelium for all four of the treatment combinations except for CXCL12+AMD3100 under both 0.50 and 2.50 dyn cm^−2^ flow conditions (Figure 6A, B). AMD3100 was able to return adhesion to baseline values at both flow rates, showing that the enhanced adhesion selectivity of circulating cancer cells is due to CXCL12 signaling through CXCR4 on the vascular endothelium (Figure 6A, B). Effects of CXCL12 are additive with enhanced adhesion mediated by treatment with TNF-α, and the CXCL12-dependent component can be inhibited with a specific chemical probe AMD3100. When 231-control cells were pre-treated with either AMD3100 or a neutralizing antibody to CXCR7 (11G8), the level of adhesion was comparable to no pre-treatment of 231-control cells onto both CXCL12 treated and untreated regions of the endothelium (p>0.34) (Figure 6C). Furthermore, the 231-control cells maintained adhesion preference to CXCL12 treated endothelium when pre-treated with AMD3100 or anti-CXCR7 antibody (p<0.05). Because inhibiting CXCR4 or CXCR7 only on 231-control cancer cells did not affect adhesion, these data further emphasize that basal treatment with CXCL12 acts through endothelial CXCR4 to significantly enhance adhesion of circulating cancer cells.

## Discussion

We have described a compact, easily-fabricated microfluidic system to model serial interactions of circulating cancer cells with a vascular endothelium at defined sites stimulated extravascularly from the basal side by chemokines. The microfluidic vasculature reproduces preferential adhesion of circulating cancer cells to endothelia in organs and tissues with high levels of CXCL12 *in vivo* [31]. This system is a substantial advancement over previously described *in vitro* flow-based assay systems in that it possesses a unique blend of: (i) prolonged, extravascular stimulation of vascular endothelium with CXCL12, modeling both the high levels and directionality of this chemokine in target organs for metastatic breast and other cancers [21] and (ii) region-specific endothelium stimulation with chemokines to enable comparison of cancer cell adhesion to endothelium of differing metastatic potential within the same experiment. Furthermore, in comparison to static assay systems, our system delivers physiologically-relevant flow conditions in micron-scale channels that reveal enhanced adhesion selectivity not observed under static conditions (Supplementary Figures S1C, Figure 4). This combination of capabilities is novel for an *in vitro* system and enables our system to evaluate the factors that mediate intravascular adhesion of circulating cancer cells in the context of physiologically defined chemical environments and physical forces.

Given its attributes, our system would also be useful for studies involving immune cell trafficking to endothelium either in response to chemokines such as CXCL12 [32] or inflammatory cytokines such as TNF-α [33]. Moreover, by integrating the principles of Transwell inserts into our microfluidic device, our system can address key differences seen in activation of endothelium from apical versus basal side stimulation but in the context of flow. For instance, it has been shown that TNF-α elicits greater leukocyte transendothelial migration across endothelium treated from the basal versus apical side [34] which suggests that spatial localization of adhesion molecules may be influenced by directionality of cytokine stimulation. We demonstrated similar results with CXCL12 by showing twice as much binding of CXCL12 and adhesion of 231-control cells onto endothelium stimulated from the basal side compared to apical (Figure 2). Since TNF-α is produced mostly by macrophages located basal or extravascular to the endothelium [35], [36], our system properly models this directionality of TNF-α stimulation which is a unique attribute compared to other *in vitro* flow-based systems. Our design also enables simultaneous basal chemokine and apical fluid mechanical stimulation of endothelia in a reagent efficient manner. Microliter amounts of expensive chemokine solution are localized to the bottom compartment while flow substantive enough to deliver shear stress levels known to alter gene expression [13], [14] can occur on the apical side of the endothelium allowing for studies that otherwise may be cost prohibitive in macroscopic or even other microfluidic systems. Furthermore, we envision our system to be a highly versatile and modular platform that can readily acquire additional physiologically important components to help provide further insights into metastasis biology. For instance, our system can be a useful co-culture model of endothelial cells with relevant supporting cells from target sites of metastasis cultured in the bottom channel. This approach is similar to what is seen in previously described macroscopic flow-based systems that integrate Transwell inserts [37], [38] but our system would retain the region-specific stimulation of the endothelium that is unique to our microfluidic system.

Using our microfluidic endothelium, we confirmed that CXCR4 or CXCR7 on breast cancer cells promotes intravascular adhesion throughout the channel (Figure 3C), supporting a mechanism through which these receptors expressed on cancer cells promote metastasis [26], [28]. These effects of CXCR4 and CXCR7 on circulating cancer cells were observed in the absence of chemokine ligand, suggesting that basal, ligand-independent activation of these seven-transmembrane receptors is sufficient to increase adhesion to endothelium. CXCR4 is known to promote cell adhesion by stimulating binding of integrins, such as α4β1 and αLβ2, to their respective ligands [39]. CXCR7 also has been shown to promote adhesion of non-transformed and cancer cells, although mechanisms of action remain to be fully defined [25], [26].

We determined that pro-metastatic effects of CXCL12 on circulating cancer cells are mediated at least in part through vascular endothelium. Stimulation of HDMECs with CXCL12 enhanced adhesion selectivity of circulating breast cancer cells by a comparable amount, regardless of expression of chemokine receptors CXCR4 or CXCR7 on cancer cells (Figure 3D). These results indicate that responses of vascular endothelium to the surrounding molecular environment contribute substantially to intravascular adhesion of cancer cells, a phenomenon difficult to address with *in vivo* studies. Furthermore, inhibiting CXCL12-CXCR4 signaling on endothelium significantly reduced adhesion of circulating breast cancer cells (Figure 6A, B) suggesting that inhibiting chemokine receptors on endothelial cells may be of equal or greater importance for preventing initial steps of intravascular cancer cell adhesion as compared with targeting these receptors on cancer cells. Endothelial cells are particularly appealing as therapeutic targets to block metastasis because these cells do not possess the same barriers to drug delivery as solid tumors [40], are readily accessible to systemic delivery of anticancer agents, and are less likely to mutate and acquire drug resistance, as compared with genetically unstable cancer cells. By combining the convenience and cost-effectiveness of *in vitro* cell culture with key physical, cellular, and molecular components of the *in vivo* vasculature, we expect that the described microfluidic endothelium will accelerate studies of the intravascular microenvironment in metastatic cancer and development of new therapies to block this key step in metastasis.

## Methods

### Device fabrication

The microfluidic device (Figure 1A) consisted of two channel layers of 12∶1 base to curing agent poly(dimethylsiloxane) (PDMS, Sylgard 184, Dow Corning) that sandwiched a semi-porous, optically clear polyester membrane [41]. The layers were sealed together using a very thin (∼10 µm) and uniform PDMS/toluene glue that provided robust, leakage-free sealing [42]. The top and bottom PDMS layers were molded as previously described [41] except the top layer featured a funnel-shaped inlet that was molded to intersect the inlet of the top channel (Figure 1A). The molded funnel-shaped inlet eliminated cell clogging that we observed in conventional cylindrical shaped inlets formed with hole punchers. This design feature enabled: a) more uniform HDMEC seeding and resulting monolayer through the entire length of the top channel and b) more consistent cancer cell suspension during the flow-based intravascular adhesion experiments (see below). The dimensions of each of the described regions (upstream, middle, and downstream) are 4,400 µm (or 4.4 mm) long by 800 µm wide. After the device was assembled, tubing was attached to the outlet of the top channel and to the inlets of the bottom channels using epoxy. Subsequently, the devices were treated with plasma oxygen (SPI Supplies) for 10 min to reduce hydrophobicity of surfaces. Immediately afterwards, the top channel was coated with a fibronectin solution (10 µg ml^−1^ PBS, 3 h, 37°C). Prior to cell seeding, the device was sterilized by placing under UV light for ∼30 min.

### Endothelial cell culture and seeding

Human dermal microvascular cells (HDMECs, Lonza) passage numbers 6–8 were cultured in EBM-2+5% FBS+SingleQuot® kit (supplements such as VEGF, bFGF, etc) or EGM-2 MV (Lonza) as described previously [43]. These cells are isolated from skin, a tissue with low metastatic potential for breast cancer [10], and facilitate low amounts of adhesion of cancer cells under non-stimulated conditions [44]. A concentrated solution of HDMECs (∼10^7^ cells ml^−1^ EGM-2 MV) was loaded through the funnel-shaped inlet reservoir, allowed to attach along the entire length of the top channel. Cells were re-fed 1–2 times per day by gravity flow from the funnel-shaped inlet and through the top channel and grown to confluence (24–48 h after seeding). We note that the presence of the bottom channels (Figure 1E), which have relatively large volumes compared to the top channel, helps guard against nutrient depletion and evaporation prone to occur in PDMS-based microchannels [45]. The concentrations for the HDMEC treatments in the described experiments were 25 µmol Syto 64, 50 ng ml^−1^ TNF-α, 100 ng ml^−1^ CXCL12, and 80 ng ml^−1^ AMD3100. These concentrations are consistent throughout (except for CXCL12-Gaussia described immediately below) and all of these treatments were in EBM-2+Single®Quots with no serum.

### Quantifying cell-associated CXCL12-Gaussia luciferase and Gaussia luciferase

293T cells stably transduced with lentiviral vectors for a fusion protein of CXCL12 and Gaussia luciferase (CXCL12-GL) or unfused Gaussia luciferase (GL) were plated in 6 cm dishes at 1×10^5^ cells per dish. One day after plating, cells were changed to DMEM medium with 0.2% bovine serum albumin (BSA) (Probumin, Celliance). Supernatants containing secreted CXCL12-GL or GL were collected from cells 18 hours later, and levels of CXCL12-GL were measured by ELISA (R&D Systems). Supernatants from CXCL12-GL secreting cells contained ≈40 ng ml^−1^ CXCL12, while this chemokine was undetectable in supernatants from GL secreting cells. CXCL12-GL has bioluminescence properties comparable to unfused GL and CXCR4-dependent signaling to a comparable extent as unfused secreted or synthetic CXCL12. Full characterization of CXCL12-GL will be described in a separate manuscript.

HDMEC cultured on 24-well Transwell inserts (Corning) were incubated for 5 hours with ≈40 ng ml^−1^ CXCL12-GL or a comparable amount of GL based on luminescence either in the top (apical) or bottom (basal) compartments of the Transwell insert. Cells and Transwell inserts were washed twice with PBS before adding 1 µg ml^−1^ coelenterazine (Fluka) in PBS and measuring bioluminescence in intact cells on an IVIS 100 system (Caliper). Images were obtained for 20–30 seconds using high resolution acquisition. Bioluminescence was quantified by region of interest analysis using Living Image Software [46] (Figure 2A).

### CXCR7 and CXCR4 expression in MDA-MB-231 human breast cancer cells

Human MDA-MB-231 breast cancer cells [47] (ATCC) were cultured in DMEM+10% FBS+1% L-glutamine+0.5% penicillin/streptomycin. Lentiviral vectors for CXCR4-GFP, CXCR7-GFP, or GFP control were used to stably transduce MDA-MB-231 cells to create the described 231-CXCR4, 231-CXCR7, or 231-control cells respectively that have native green fluorescence for clear detection. Chemokine receptor expression in the 231-CXCR4, 231-CXCR7 cells, and 231-control cells was verified by flow cytometry (Figure 3A) as we have described previously, using mouse monoclonal antibodies to CXCR4 (12G5-PE, R&D Systems) or CXCR7 (11G8-APC, gift of R&D Systems) [48].

### RT-PCR

Total RNA from HDMECs (Figure 5A) was prepared using Trizol reagent (Invitrogen) according to the manufacturer's protocol. RNA was purified further over an RNA extraction column (Qiagen), including on-column treatment with DNaseI. RT-PCR was performed using a two-step kit (ThermoScript, Invitrogen). Sequences of PCR primers were the following:

CXCR4: 5′-ACGGACAAGTACAGGCTGCAC-3′ and 5′-CCCAGAAGGGAAGCGTGA-3′

CXCR7: 5′-AAGAAGATGGTACGCCGTGTCGTCTC-3′ and 5′-CTGCTGTGCTTCTCCTGGTCACTGGA-3′

GAPDH: 5′-GAAGGTGAAGGTCGGAGT-3′ and 5′-GAAGATGGTGATGGGATTTC-3′

### Western Blot

HDMECs were incubated with 100 ng ml^−1^ CXCL12 for 10 minutes or 5 hours. Control cells were incubated with BSA alone. 231 breast cancer cells were cultured overnight in DMEM medium containing 0.5% serum. Breast cancer cells then were treated with 100 ng ml^−1^ CXCL12 (R&D Systems) for 3 or 10 minutes, respectively (Figure 5B). Total cell lysates were harvested and prepared for Western blotting as described previously [49]. Primary antibodies to AKT phosphorylated at serine 473 and total AKT (Cell Signaling Technology) were used at 1∶500 dilution, and a secondary antibody to rabbit conjugated with horseradish peroxidase was used at 1∶2000 dilution. Western blots were developed with ECL reagent (Amersham).

### Calculation of shear stress levels

The shear stress levels on the cells within the channels were modeled with the following equation [50], [51]:
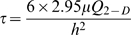
(1)Where *τ* is the shear stress level at the cell apex, *μ* is the viscosity of the fluid (water = 0.01 dyn·s cm^−2^ at room temperature), *Q_2-D_* is the flow rate per width in the system and *h* is the height of the channel. This equation is valid for cases where the width is much larger than the height. In our system, the height of the channels was 60 µm and the width was 800 µm.

### Flow-based intravascular adhesion experiments

Prior to each experiment, the EGM-2 MV culture in the funnel-inlet of the microfluidic device was removed, washed with PBS, and replaced with EBM-2+Single®Quots. Subsequently, the top channel was washed with EBM-2+Single®Quots by gravity-driven flow. Concurrently, the bottom channels were triple washed with PBS and replaced with either EBM-2+Single®Quots (untreated) or EBM-2+Single®Quots+cytokine or chemokine of interest. The flow in the top channel was stopped and the device was left to be treated for 5 h. Concurrently, culture flasks of the 231 breast cancer cells were washed with PBS and serum starved in EBM-2+0.5% FBS for 5 h as well. For experiments involving pre-treatment of 231 cells with chemokine receptor inhibitor (Figure 6C), either AMD3100 (80 ng ml^−1^) for neutralizing CXCR4 or 11G8 antibody (10 µg ml^−1^) for neutralizing CXCR7 is added to the EBM-2+0.5% FBS starve media.

After serum starvation, the breast cancer cells were collected using citric saline, a calcium ion chelator, instead of trypsin to ensure cell surface proteins remain intact. The cancer cells were centrifuged and washed twice and resuspended in EBM-2+0.5% FBS at a concentration of 2×10^6^ cells ml^−1^. The cancer cell suspension was loaded into the funnel-shaped inlet. Flow was controlled using a programmable syringe pump (KD Scientific) that withdraws fluid away from the funnel-shaped inlet at flow rates corresponding with shear stress levels of 0.50 dyn cm^−2^ or 2.50 dyn cm^−2^. We note that in our system, the shear stress levels of 0.50 dyn cm^−2^ and 2.50 dyn cm^−2^ correspond with flow velocities of 0.2 mm s^−1^ and 1.0 mm s^−1^ respectively which are in line with the *in vivo* blood flow velocity range of 0.1–1.5 mm s^−1^ reported in the microcirculation of potential sites of breast cancer metastasis [52]–[54]. Also, integrins on the surface of circulating cells are reported to optimally mediate adhesion onto endothelium at shear stress levels below 0.50 dyn cm^−2^ with the avidity decreasing rapidly with higher shear stress levels [18]. Thus, the absolute number of cells attached is much lower at 2.50 dyn cm^−2^ compared to 0.50 dyn cm^−2^.

The microfluidic device was placed on the stage of an epi-fluorescence, inverted microscope (Nikon, TE-300). The duration of each experiment was 30 min followed by a controlled 1 min wash of the top channel with PBS to remove loosely attached cells. Images were recorded with a digital CCD camera (Hamamatsu). To determine cell counts, fluorescent cells in an entire region were counted blindly by a different person other than the experimentalist to negate observer bias using a semi-automatic counting protocol with NIH ImageJ and treated as one data point.

### Static adhesion experiments

The conditions for the static adhesion experiments were the same as the flow-based experiments described above in terms of cells, pre-treatment, and duration of experiments. The difference was the static experiments were performed in tissue culture 96-well plates (Corning) instead of microfluidic devices. Into each well, ≈10,000 cancer cells in 100 µl were added and allowed to adhere for 30 minutes. Following the adhesion experiments, each well was triple washed with PBS to remove loosely attached cells. For static adhesion in Transwells (Figure 2B), HDMECs were grown to confluence in 24-well plate Transwells similar to the CXCL12-GL binding assays (Figure 2A). The adhesion experiments were performed in the same manner as the 96-well plate experiments described above with CXCL12 (100 ng ml^−1^) introduced either apical or basal to the endothelium for 5 hours. Also, the same amount of cancer cells and volume (10,000 cells in 100 µl) were allowed to adhere since the surface area of the Transwell insert is the same as an individual well in a 96-well plate (0.32–0.33 cm^2^). In these experiments, 10 minutes instead of 30 minutes were allowed for adhesion prior to triplicate PBS washing.

### Statistical analysis

Sample populations were compared either pair wise using the Mann-Whitney U test or in groups of more than two using the Kruskal-Wallis test. p<0.05 was the threshold for statistical significance. Data sets that were statistically significant with the Mann-Whitney U test were indicated with a ‘*’ symbol. For data evaluated with the Kruskal-Wallis test, results that were statistically significant were indicated with a ‘**’ symbol; results that were not statistically significant were indicated with a ‘#’ symbol.

## Supporting Information

Figure S1 231-control cells preferentially adhere onto TNF-α stimulated endothelial region at 0.50 and 2.50 dyn cm^−2^ flow conditions. TNF-α was applied to either the upstream (black bars) or downstream compartment (white bars) with the other compartments in the same device left untreated. Cancer cell adhesion onto the TNF-α treated endothelial region was significantly greater than the untreated region regardless of if TNF-α was applied upstream or downstream (p<0.01). (A) 0.50 dyn cm^−2^. (B) 2.50 dyn cm^−2^. ‘+’ denotes TNF-α stimulation. n = 3 each for upstream or downstream treated conditions. Data are expressed as the mean+s.e.m. (C) Boxplots representing adhesion selectivity of 231-control cells towards TNF-α treated endothelium at 0, 0.50, and 2.50 dyn cm^−2^ shear stress levels. The adhesion selectivity was statistically different for the different flow conditions (**, p<0.05) with the selectivity increasing with increasing flow. n = 6 for each condition.
(0.26 MB TIF)
