# Supplementary figures and images for: Microfluidic Endothelium for Studying the Intravascular Adhesion of Metastatic Breast Cancer Cells
Source: PLoS One. 2009 Jun 1;4(6):e5756. doi: 10.1371/journal.pone.0005756 (PMC2684591; doi:10.1371/journal.pone.0005756)

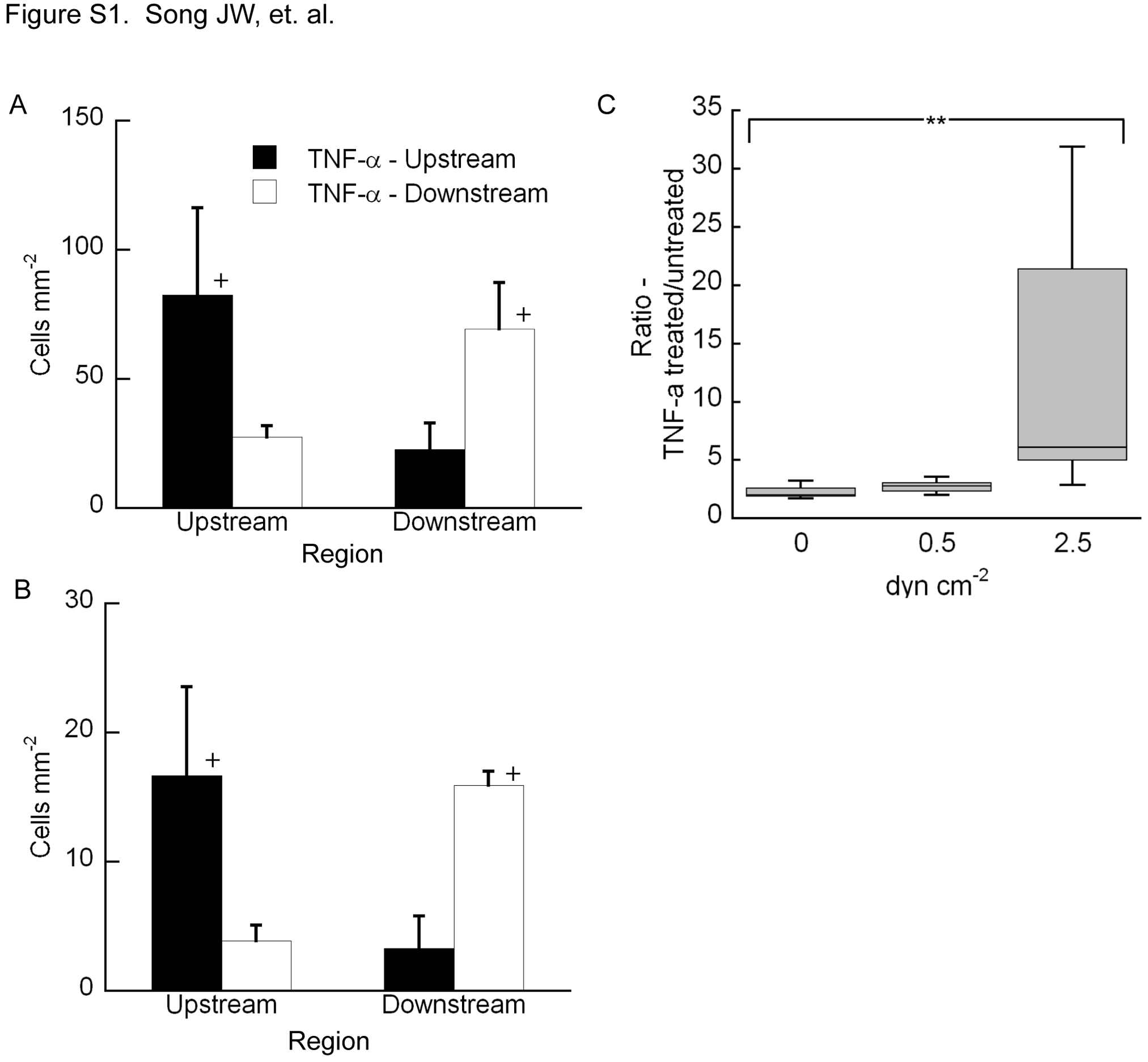

Supplement: Figure S1 — 231-control cells preferentially adhere onto TNF-α stimulated endothelial region at 0.50 and 2.50 dyn cm−2 flow conditions. TNF-α was applied to either the upstream (black bars) or downstream compartment (white bars) with the other compartments in the same device left untreated. Cancer cell adhesion onto the TNF-α treated endothelial region was significantly greater than the untreated region regardless of if TNF-α was applied upstream or downstream (p<0.01). (A) 0.50 dyn cm−2. (B) 2.50 dyn cm−2. ‘+’ denotes TNF-α stimulation. n = 3 each for upstream or downstream treated conditions. Data are expressed as the mean+s.e.m. (C) Boxplots representing adhesion selectivity of 231-control cells towards TNF-α treated endothelium at 0, 0.50, and 2.50 dyn cm−2 shear stress levels. The adhesion selectivity was statistically different for the different flow conditions (**, p<0.05) with the selectivity increasing with increasing flow. n = 6 for each condition. (0.26 MB TIF) [file pone.0005756.s001.tif]
